# 1379. Evaluation of Cardiovascular Disease Risk among People Living with HIV in Lebanon

**DOI:** 10.1093/ofid/ofad500.1216

**Published:** 2023-11-27

**Authors:** Remie El Helou, Mariam Zaidan, Saliba Wehbe, Nesrine Rizk

**Affiliations:** American University of Beirut Medical Center, Beirut, Beyrouth, Lebanon; American University of Beirut, Beirut, Beyrouth, Lebanon; American University of Beirut Medical Center, Beirut, Beyrouth, Lebanon; American University of Beirut, Beirut, Beyrouth, Lebanon

## Abstract

**Background:**

Since the introduction of antiretroviral therapy (ART), People Living with HIV (PLHIV) have become more prone to developing chronic illnesses, such as cardiovascular diseases (CVD), than to suffer from AIDS related conditions. The global burden of CVD associated with HIV has tripled over the last two decades, especially in low/middle income countries. Scarce data exist on the prevalence of CVD and CVD risk factors among PLHIV in the Middle East and North Africa (MENA) region. Therefore, we aim to examine the prevalence of CVD risk factors and calculate the CV risk in a cohort of PLHIV in Lebanon.

**Figure:**
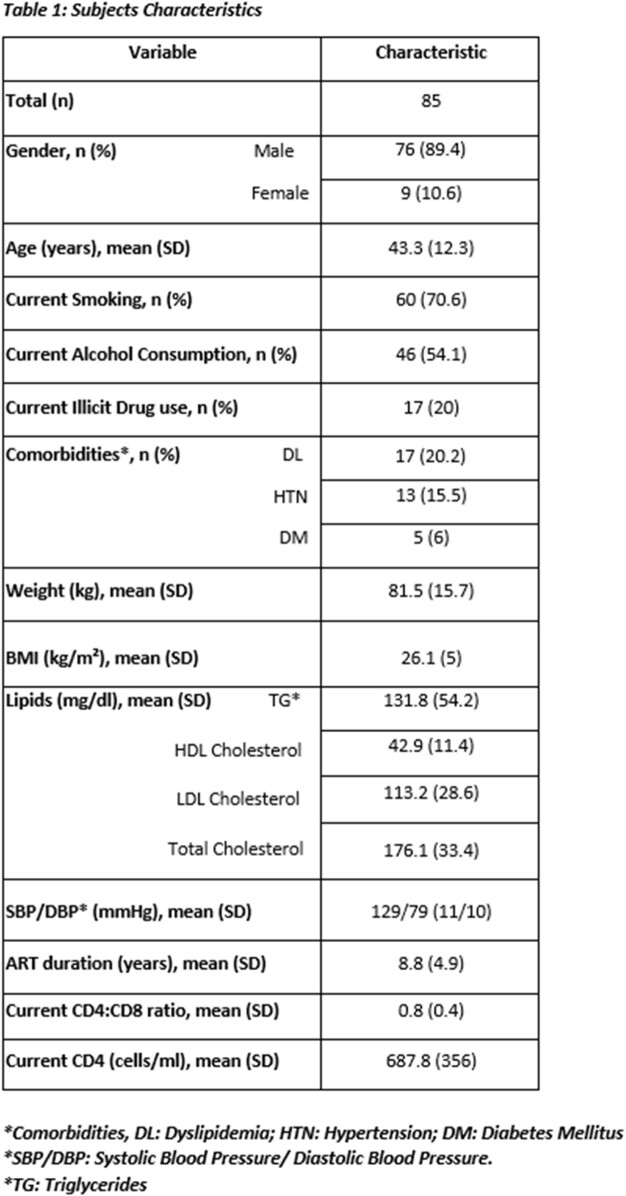

**Methods:**

This is a retrospective chart review conducted between 2018 and 2023 at an HIV clinic in a tertiary care center in Lebanon. The study population includes adult subjects receiving ART and with at least 6 months of undetectable viral loads. Data included demographics, laboratory values, HIV-specific data, and review of traditional CVD risk factors. Atherosclerotic Cardiovascular Disease (ASCVD) risk score was calculated using the American College of Cardiology Risk Estimator Plus, to estimate patients’ 10-year risk for developing ASCVD. Descriptive statistics were implemented to summarize the cohort’s characteristics and the prevalence of CVD risk factors.

**Results:**

A total of 85 HIV-infected subjects were included in the analysis. Subjects’ characteristics are summarized in table 1. The majority were males (89.4%), and the mean age was 43.31±12.28 years. The prevalence of smoking, alcohol consumption, and illicit drug use was 70.6%, 54.1%, and 20%, respectively. The mean BMI was 26.1±5 kg/m². Additionally, 20.2 % of subjects had dyslipidemia, 6% had diabetes, and 15.5% had hypertension. The mean current CD4 cell count was 687.83 ±356 cells/ml. The cohort had a mean ASCVD risk score of 8.8%, higher than that of age-matched population (mean ASCVD risk score of US firefighters aged 40-44 years is 1.8%).

**Conclusion:**

This study shows the prevalence of traditional CVD risk factors among PLHIV receiving effective ART in Lebanon. The cohort mean ASCVD risk score indicates an intermediate risk for developing CVD over the next decade. in the MENA region, we need larger studies to inform on the prevalence of CVD among PLHIV, and to develop guidelines to mitigate the risk.

**Disclosures:**

**All Authors**: No reported disclosures

